# Analysis of the related factors of atypical squamous cells of undetermined significance (ASC-US) in cervical cytology of post-menopausal women

**DOI:** 10.3389/fcimb.2023.1123260

**Published:** 2023-02-16

**Authors:** Bijun Li, Lichang Dong, Chen Wang, Jia Li, Xue Zhao, Mengting Dong, Huanrong Li, Xiaotong Ma, Yalan Dong, Ming Wu, Ye Yan, Aiping Fan, Fengxia Xue

**Affiliations:** ^1^ Department of Gynecology and Obstetrics, Tianjin Medical University General Hospital, Tianjin, China; ^2^ Tianjin Key Laboratory of Female Reproductive Health and Eugenic, Department of Gynecology and Obstetrics, Tianjin Medical University General Hospital, Tianjin, China

**Keywords:** cervical cytology, post-menopausal women, atypical squamous cells, cervical intraepithelial neoplasias, vaginal microecology

## Abstract

**Introduction:**

Atrophy of the reproductive tract mucosa caused by the decrease of estrogen may increase the detection rate of ASC-US in cervical cytology of post-menopausal women. In addition, other pathogenic infections and inflammation can change the cellular morphology and increase the detection rate of ASC-US. However, further studies are needed to elucidate whether the high detection rate of ASC-US in post-menopausal women leads to the high referral rate of colposcopy.

**Methods:**

This retrospective study was conducted to document ASC-US in cervical cytology reports at the Department of Cytology at Gynecology and Obstetrics, Tianjin Medical University General Hospital between January 2006 and February 2021. We then analyzed 2,462 reports of women with ASC-US at the Cervical Lesions Department. A total of 499 patients with ASC-US and 151 cytology with NILM participants underwent vaginal microecology tests.

**Results:**

The average reporting rate of ASC-US in cytology was 5.7%. The detection rate of ASC-US in women aged > 50 years (7.0%) was significantly higher than that in women aged ≤50 years (5.0%) (P<0.05). The CIN2+ detection rate was significantly lower in the post- (12.6%) than in pre-menopausal (20.5%) patients with ASC-US (P <0.05). The prevalence of abnormal reporting rate of vaginal microecology was significantly lower in the pre-menopausal group (56.2%) than that in the post-menopausal group (82.9%) (P<0.05). The prevalence of bacterial vaginosis (BV) (19.60%) was relatively high in the pre-menopausal group, but the abundance of bacteria-inhibiting flora (40.79%) was mainly an abnormality in the post-menopausal group. The vaginal microecological abnormality rate of the women with HR-HPV (-) of ASC-US was 66.22%, which was significantly higher than that of the HR-HPV (-) and the NILM group (52.32%; P<0.05).

**Discussion:**

The detection rate of ASC-US in women aged > 50 years was higher than that ≤50 years, but the detection rate of CIN2+ was lower in the post-menopausal women with ASC-US. However, vaginal microecological abnormalities may increase the false-positive diagnosis rate of ASC-US. The vaginal microecological abnormalities of the menopausal women with ASC-US are mainly attributed to infectious diseases such as BV, and it mainly occurs in the post-menopausal women was bacteria-inhibiting flora. Therefore, to avoid the high referral rate for colposcopy, more attention should be paid to the detection of vaginal microecology.

## 1 Introduction

Cervical cancer remains a significant public health concern among women in China. The National Cancer Center (NCC) of China reported in the 2016 nationwide statistics for cancer that the incidence and mortality rates of cervical cancer were ranked the fifth and seventh among all the female malignancies, respectively, and these rates continue to be on the rise (Zheng et al., 2022). Therefore, selecting more effective screening methods and improving the diagnosis rate of cervical precancerous lesions are of great significance in accelerating the elimination of cervical cancer. Atypical squamous cells (ASC) are the most prevalent form of all the abnormal cervical cytology interpretations (Nayar and Wilbur, 2015). However, the management of women with ASC-US remains a clinical challenge because of the variable underlying processes from human papillomavirus (HPV)-unrelated, non-neoplastic conditions to HPV-related cervical intraepithelial neoplasia (CIN) and carcinomas (Solomon et al., 2002). ASC-US is equivocal and the diagnosis is highly subjective. In addition to HPV infection and tumor-related factors, other pathogenic infections, inflammation, atrophy, intrauterine device (IUD), and air dryness can change the cellular morphology and may lead to the misdiagnosis of ASC-US (Watson et al., 2015; Bruno et al., 2019; Koh et al., 2019).Continuous infection with high-risk human papillomavirus (HR-HPV) is necessary for the development of cervical cancer, which is clearly associated with abnormal cytology. In recent years, many countries, including the United States, have recommended triage HPV testing after cytology showing ASC-US and colposcopy for all HR-HPV-positive cases (Zeferino et al., 2018; Kyrgiou et al., 2020; Perkins et al., 2020; Bhatla et al., 2020). These strategies may be associated with excessive care (Zeferino et al., 2018; Kyrgiou et al., 2020; Perkins et al., 2020; Bhatla et al., 2020). First, the distribution of HR-HPV varies geographically across continents, particularly HPV52 and HPV58, whose prevalences are notably higher in cases of invasive cervical cancer in China than those of HPV45 and HPV18 (Zhou et al., 2018). Second, based on the different carcinogenicities of HPV genotypes, the HPV genotype has various impacts on the development of cervical cancer and CIN. It has been reported that women with ASC-US and HPV16 or HPV18 positive are more likely to develop CIN3+ than women with other HR HPV types (Stoler et al., 2011). It has also been reported that women with ASC-US with HPV16, 18, 31, 33, or 58 genotypes are most likely to develop CIN2 (Wang et al., 2021). As a result, type-specific HPV identification appears to be a reasonable strategy for reducing the colposcopy burden in women with ASC-US and improving the risk stratification. In addition, the transformation zone of post-menopausal women moved up, squamous metaplasia was slow, immunity declined, and the lower reproductive tract was more susceptible to carcinogenic factors, which facilitated the development of persistent infection (Brotman et al., 2014). Studies have shown that the rate of cytological abnormalities in the women over 50 years of age is dramatically higher than that in the women aged < 50 (Zheng et al., 2019; Wang et al., 2020b; Tao et al., 2021). However, it is still debatable whether there is a difference in the prognosis between pre- and post-menopausal women. It has been reported that the detection rate of CIN2+ in women with ASC-US over 50 years of age is much higher than that under 50 years of age (Wang et al., 2020b). In one survey, the incidence of CIN3+ lesions was higher in women younger than 30 years and those older than 60 years old (Tai et al., 2018). However, some studies have suggested that there is no difference in the HSIL+ detection rate in women with ASCUS before and after 50 years old (Abdulaziz et al., 2020). Therefore, the relationship between ASC-US and histopathology in post-menopausal women remains unclear and requires further investigation.

In addition to HPV infection, other synergistic factors also affect the cervical cytology results. Bacterial vaginosis (BV), vulvovaginal candidiasis (VVC), trichomoniasis and aerobic vaginitis (AV) are associated with an increased risk of CIN (Peres et al., 2015; Plisko et al., 2021). Liu et al. reported that the positive rate of HR-HPV increased with the cytological severity, but vaginal microecological abnormalities were mainly related to ASC-US and LSIL (Liu et al., 2021). Some other studies have also revealed that vaginal microecological abnormalities are associated with ASC-US (Gomes de Oliveira et al., 2018). In addition, it has been reported that post-menopausal women are prone to microecological disorders, such as bacterial community disorder and impaired vaginal immune barrier due to decreased estrogen levels, atrophy and thinning of vaginal mucosa, decreased glycogen content in epithelial cells, and the decrease in the species of *Lactobacillus* (Xu et al., 2021). It has also been confirmed that the “atrophic” form caused by the decreased hormone levels can increase the detection rate of ASC-US in post-menopausal women (Cakmak and Köseoğlu, 2014).

In the current study, we document and analyze the reporting rates of the age-stratified ASC-US, the immediate histopathology of ASC-US along with HR-HPV genotype distribution, outcomes of post-menopausal women with ASC-US, and the correlation between vaginal microecology and ASC-US from the department of Gynecology and Obstetrics, Tianjin Medical University General Hospital. This aims at offering new parameters to design an optimal strategy for the triage of pre- and post-menopausal patients with ASC-US.

## 2 Materials and methods

### 2.1 Study population

**Figure 1:**
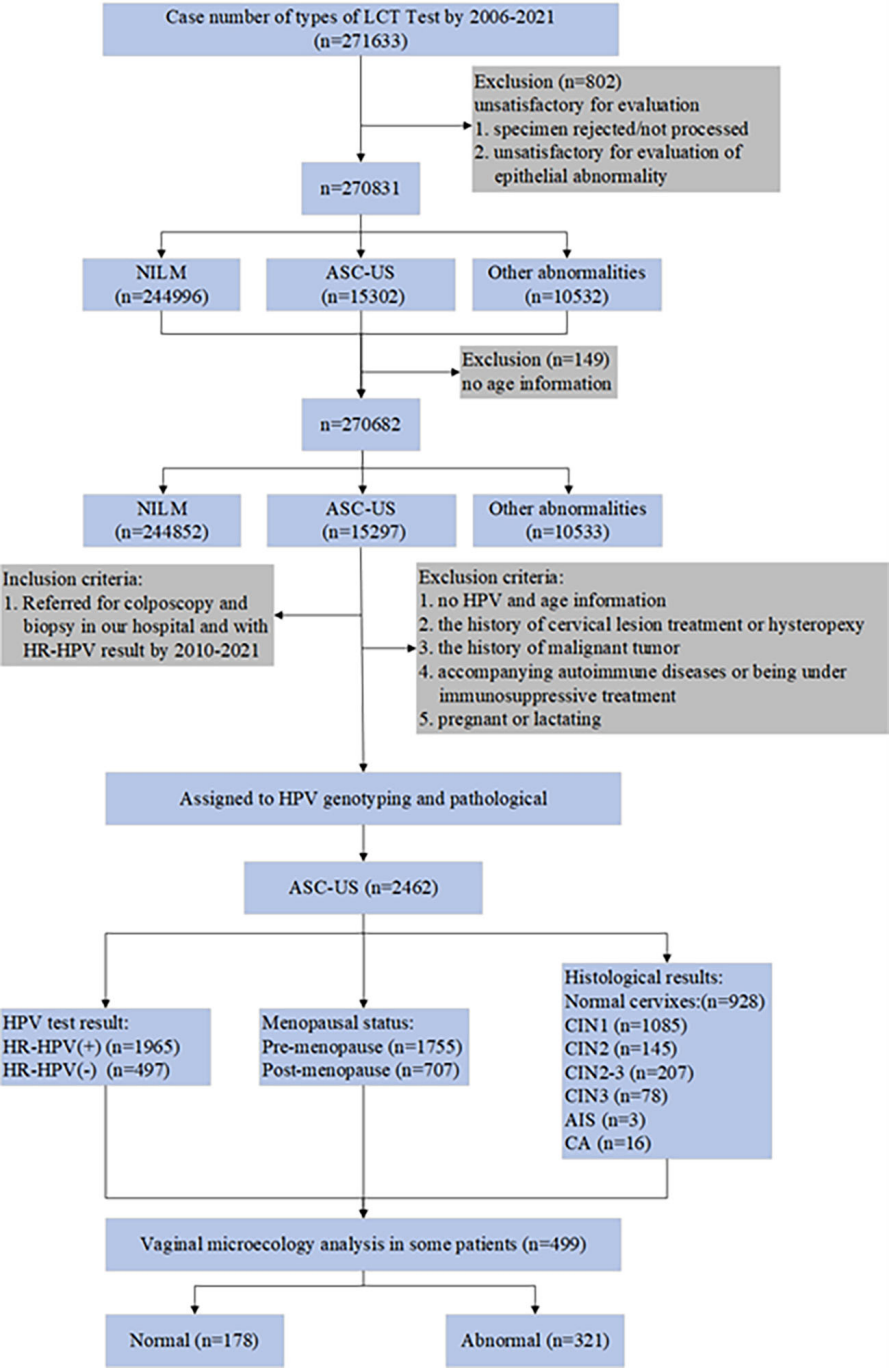
The flowchart of the study.

This retrospective study was conducted to document the cervical cytology reports of patients with ASC-US at the Department of Cytology at Gynecology and Obstetrics, Tianjin Medical University General Hospital (TMUGH) between January 2006 and February 2021. We then analyzed 2,462 reports of women with ASC-US who underwent HPV genotyping, colposcopy, and histopathological examination at the Cervical Lesions Department of Obstetrics and Gynecology between January 2010 and December 2021. It is important to emphasize that a total of 499 participants with ASC-US and 151 with HR-HPV-negative and NILM cytology underwent vaginal microecology tests. The inclusion and exclusion criteria are shown in **Figure 1**. 2462 patients underwent colposcopy and biopsy. The menstrual status data were available for 2,018 women, while the information on the menstrual status was unknown for 444 women. Of the 2,018 women, 507 were confirmed as post-menopausal. The mean age of the post-menopausal women was 50.23 years. Using the previously reported age cut-off recommendations to classify menopause (Holt et al., 2017), the mean age of our study was then used as a cut-off to define the post-menopausal status in the remaining 444 women. With this classification, an additional 197 women were included in the post-menopausal group, resulting in a total number of 707 women in the studied menopausal group and 1,755 in the pre-menopausal group.

### 2.2 Cytology testing

The Liquid-based cytological test (LCT) method was used in this study. LCT preparation was performed by Becton, Dickinson and Company. Cytological evaluation was performed by two cytopathologists at the Department of Cytology at Gynecology and Obstetrics, TMUGH. Cytological results were classified using the 2001 TBS criteria for reporting cervical cytology before 2014 (Solomon et al., 2002), and the rest were classified using the 2014 TBS criteria (Nayar and Wilbur, 2015). A cytopathologist who was blinded to the results reviewed the cases with an ASC-US interpretation or worse.

### 2.3 HPV genotyping

The HR-HPV genotypes (16,18,31,33,35,39,45,51,52,53,56,58,59,66,68,73,82 and 83) were detected using a polymerase chain reaction-reverse dot blot (PCR-RDB) HPV genotyping kit (YaNeng Biosciences, Shenzhen, China). The procedures were performed in accordance with the manufacturer’s instructions. A small number of patients were tested for HPV in other hospitals, and the HPV test was performed using the Roche Cobas 4800 HPV DNA and HC2 tests. The Roche Cobas 4800 HPV DNA test can detect 14 HR-HPV types, including HPV16,18,31,33,35,39,45,51,52,56,58,59,66 and 68. HC2 test can be used for the quantitative detection of 13 high-risk HPV types, without HPV genotype, and ≥1.0pg/mL is considered positive.

### 2.4 Colposcopy and histological examination

ASC-US with positive HR-HPV or negative HR-HPV and more than twice ASC-US cytology were referred for colposcopy for biopsy. Colposcopy and biopsy were performed by experienced gynecological specialists from TMUGH. The duration between cytological sampling and colposcopy did not exceed 60 days. During the colposcopy, all the visually abnormal areas were biopsied. Quadrants with normal colposcopic impressions were biopsied at the squamocolumnar junction (“random biopsy”). Endocervical curettage(ECC)is performed when the TZ is not visible or fully visible. All histological slides were reviewed by two gynecological pathologists who were blinded to the cytology results at TMUGH. Immunohistochemistry was used to adjudicate difficult or equivocal diagnoses.

### 2.5 Vaginal microecology analysis

Women with suspicious vaginitis, uncomfortable symptoms, or routine physical examination underwent vaginal microecological analysis before and after the cytology test. An unlubricated sterile speculum was inserted before any other vaginal examinations were performed. Sterile long cotton swabs were used to obtain the vaginal discharge from the upper lateral vaginal wall for vaginal pH measurements (special indicator paper, pH 3.8–5.4; Shanghai SSS Reagent Co. Ltd., Shanghai, China) and microscopic examinations. Vaginal discharge smears were spread on 3 glass slides for immediate microscopic examination. AV, BV, Trichomoniasis, and VVC were defined according to their recognized standards. The Nugent score was used to diagnose BV and was calculated by assessing the number of *Lactobacillus* morphotypes (scored as 0–4), *G. vaginalis* morphotypes (scored as 0–4), and *Mobiluncus* morphotypes (scored as 0–2). A Nugent score of 7–10 was interpreted as consistent with BV, a score of 4–6 as intermediate (BV intermediate type), and a score of 0–3 as negative for BV. Wet-mount microscopy has traditionally been used as the preferred diagnostic test for trichomoniasis in women. A diagnosis of Candida vaginitis is indicated by a wet preparation (saline, 10% KOH) of vaginal discharge demonstrating budding yeasts, hyphae, or pseudohyphae (Workowski et al., 2021). The diagnosis of aerobic vaginitis (Donders et al., 2017) by microscopic examination included the evaluation of lactobacillary grades (LBG) (scored as 0–2), the number of leukocytes (scored as 0–2), the proportion of toxic leukocytes (scored as 0–2), the type of background bacteria (score 0–2), and the number of parabasal epithelial cells under the wet film using a phase-contrast microscope (×400, score 0–2). An AV score ≥3 indicated AV. Two or more types of pathogenic microorganisms coexisting in the vagina were diagnosed as mixed vaginitis. Examination of atrophic vaginitis revealed atrophy of the external genitalia, along with loss of the vaginal rugae. Vaginal mucosa may be friable in some areas. Microscopy of vaginal secretions showed a predominance of parabasal epithelial cells and an increased number of leukocytes. The bacteria-inhibiting flora was significantly reduced, showing no dominant bacteria with a density grade ≤ I and a diversity grade ≤ I. After excluding other types of vaginitis, a mean WBC count of ≥10/HPF in five discontinuous fields at 400 wet preparations was diagnosed as leukocytosis (Cooperative Group of Infectious Disease, Chinese Society of Obstetrics and Gynocology, Chinese Medical Association, 2016). The results of vaginal microecology detection were diagnosed by experienced physicians in the Microecology Department of Obstetrics and Gynecology of TMUGH.

### 2.6 Statistical analysis

The statistical analysis was performed using the Statistical Package for the Social Sciences (SPSS) version 25.0 software for windows (SPSS Inc., Chicago, IL, USA). Chi-square test or Fisher’s exact test was used for analyzing categorical variables. Statistical tests were two-sided, and *P* < 0.05 was considered statistically significant. Cochran and Mantel-Haenszel tests were performed to evaluate the common odds ratio (OR) of the HPV-positive group compared with the HPV-negative group, controlled by pre- and post-menopause. A *P-*value < 0.05 was considered statistically significant.

## 3 Results

### 3.1 ASC-US reporting rates

During the 16-year retrospective study period, a total of 270,682 gynecologic LCT tests were performed (148 patients have been excluded due to the lack of their age-related information). ASC-US cytology was the most frequent abnormal interpretation, accounting for 5.7% of all cervical cytology in the survey (**Table 1**). The detection rate of ASC-US in women aged >50 years (7.0%) was significantly higher than that in women aged ≤50 years (5.0%; *P <*0.05). The ASC-US reporting rates of 7.3%, 7.1%, and 7.1% of in the age groups of 51–60 years, 61–70 years, and below 20 years, respectively, were significantly higher than the reporting rates of 5.4%, 5.1%, 4.9%, 4.5%, and 4.4% observed in the age groups of 41–50 years,71–80 years, 31–40 years, 21–30 years, and > 80 years, respectively (*P* < 0.05).

**Table 1:** Numbers of Cervical cytology result in different age groups.

Age	No. of cervical cytology result						
	Total (No.)	NILM (No, %)		Abnormalities (No, %)		ASC-US (No, %)	
≤20	1695	1451	85.6	244	14.4	120	7.1
21-30	36035	32993	91.6	3042	8.4	1610	4.5
31-40	80710	73899	91.6	6811	8.4	3983	4.9
41-50	67378	61057	90.6	6321	9.4	3646	5.4
51-60	51823	45822	88.4	6001	11.6	3767	7.3
61-70	25173	22398	89.0	2775	11.0	1775	7.1
71-80	6835	6273	91.8	562	8.2	351	5.1
>80	1033	959	92.8	74	7.2	45	4.4
Total (No.)	270682	244852	90.5	25830	9.5	15297	5.7

### 3.2 Histopathologic results and HPV test results for cases with ASC-US

A total of 2,462 eligible patients had confirmed ASC-US by cytology and were included in the study between January 2010 and December 2021, with an average age of 43.06 ± 12.27 years (range, 18–85 years). The overall HPV test results revealed that 2,381 patients had the HPV genotype, 39 had Cobas 4800, and 42 had HC2. Ultimately, there were 1,965 patients with HPV-positive status and 497 patients with HPV-negative status. The histological analysis confirmed that 928 patients (37.7%) had normal cervixes, 1,085 (44.1%) had CIN1, 145 (5.9%) had CIN2, 207 (8.4%) had CIN2–3, 78 (3.2%) had CIN3, 3(0.1%) had AIS, and 16 (0.6%) had carcinoma (including 15 squamous cell carcinomas and 1 adenocarcinoma). Among the 2,462 patients with histopathological results, diagnoses of CIN2+ were reported in 449 patients (18.2%).

The CIN2+ detection rate was significantly lower in the post- than in the pre-menopausal patients with ASC-US, as demonstrated in **Table 2** (12.6 vs. 20.5%, respectively;X^2 =^ 21.224, *P*=0.000, <0.05).

**Table 2:** The histopathologic results for pre- and post-menopausal women with ASC-US.

		No. of Cases (%)		
	Average age	≤CIN1	CIN2+	Total
Pre-menopause	37.02	1395(79.50)	360(20.50)	1755
Post-menopause	58.31	618(87.4)	89(12.60)	707
***P***		0.00		

CIN2+ lesions were found in 21.5% (422 of 1,965) of the women with ASC-US/HPV-positive results which is significantly higher than the rate of 5.4% for women (27 of 497) with ASC-US/HPV-negative results (X^2 =^ 68.470, *P*=0.000, <0.05). (**Table 3**).

**Table 3:** The histopathologic results for positive and negative HPV women with ASC-US.

		No. of Cases (%)		
		≤CIN1	CIN2+	Total
HPV	Positive	1543(78.5)	422(21.5)	1965
	Negative	470(94.6)	27(5.4)	497
*P*		0.00		

In the patients with ASC-US/HR-HPV-positive status, the CIN2+ detection rate was significantly lower in the post- than in pre-menopausal patients with ASC-US, as demonstrated in **Table 3** (15.0 vs. 24.0%; *P <*0.05). Furthermore, in the patients with ASC-US/HR-HPV-negative status, there was no significant difference in the detection rate of CIN2+between the post- and pre-menopausal women (X^2 =^ 0.514, P=0.474,*P >*0.05). The detection rates of CIN2+ in the HR-HPV-positive group were significantly higher than those in the HPV-negative group in both the pre-menopausal or post-menopausal women with ASC-US (all *P<*0.05; **Table 4**). In addition, Cochran and Mantel–Haenszel tests were performed to evaluate the common OR of the HR-HPV-positive group compared with the HPV-negative group in predicting the CIN2+ lesions when the menopausal status was controlled. The common OR = 4.687 (95%CI, 3.133–7.013; *P*=0.00).

**Table 4:** Histopathologic results for pre- and post-menopausal women with ASC-US and HPV test results.

	No. of negative HPV Cases (%)			No. of positive HR- HPV Cases (%)		
	≤CIN1	CIN2+	Total	≤CIN1	CIN2+	Total
Pre-menopause	317(94.1)	20(5.9)	337	1078(76.0)	340 (24.0)^a^	1418
Post-menopause	153(95.6)	7(4.4)	160	465(85.0)	82(15.0)^a^	547
Total	470(94.6)	27(5.4)	497	1543(78.5)	422(21.5)	1965
*P*	0.474			0.000		

a P < 0.05 compared with the HPV-positive counterpart groups.

A total of 475 (19.6%) patients were HPV16 positive, 147 (6.1%) were HPV18 positive, 25 (1.0%) were HPV16 and HPV18 positive, and 1,276 (52.7%) were HR HPV-positive. HPV16, HPV16 and 18 positive both showed the severity of the histological diagnosis, and the rate of CIN2+ was significantly higher than the HPV18 positive group and the HR HPV-positive group (*P <*0.05). Furthermore, the CIN2+ detection rate of the HPV16/18 positive patients with ASC-US was significantly higher than that of the other HR HPV-positive groups, *P* < 0.05. The detailed results for the pre-and post-menopausal women with ASC-US are provided in **Table 5**. The CIN2+ detection rates in the pre-menopausal patients with ASC-US in the HPV16 positive group, other HR HPV-positive group, and HPV16/18 positive group were significantly higher than that in the post-menopausal patients, *P* < 0.05. However, the detection rates of CIN2+ in the pre- and post-menopausal HPV18 positive group were similar (*P >*0.05).

**Table 5:** Histopathologic results for pre- and post-menopausal women with ASC-US according to HPV genotyping.

		No. of pre-menopause Cases (%)			No. of post-menopause Cases (%)		
		≤CIN1	CIN2+	Total	≤CIN1	CIN2+	Total
negative		317(94.1)	20(5.9)	337	153(95.6)	7(4.4)	160
HPV16/18	HPV16	201(56.6)	154(43.4)	355	83(69.2)	37(30.8)	120
	HPV18	95(81.2)	22(18.8)	117	26(86.7)	4(13.3)	30
	HPV16+18	8(47.1)	9(52.9)	17	6(75.0)	2(25.0)	8
other types		747(83.7)	146(16.3)	893	345(90.1)	38(9.9)	383
Total		1368(79.6)	351(20.4)	1719	613(87.4)	88(12.6)	701

In the patients with ASC-US with a single infection, HPV16 was the most common HR-HPV genotype, followed, in order of decreasing frequency, by HPV52, 58, 18, and 33. The detection rate of CIN2+ in HPV16 (40.9%) was similar to that of HPV33 (29.9%; *P* > 0.05), but significantly higher than that of HPV52, 58, and 18 (*P* < 0.05). The detection rate of the CIN2 + lesions from the five most common HR-HPV genotypes (HPV16, 52, 58, 18, and 33) in women was 25.6% (227/885), which was significantly higher than that of the other single HR-HPV types (6.8%, 30/439, *P* < 0.05).

The single HPV-positive results in the pre-menopausal patients with ASC-US were as follows: HPV16, 52, 58, 18, 33, and 53. In the pre-menopausal women, the order was slightly similar.

The detection rates of CIN2+ in HPV16, 58, 18, and 33 in the pre- and post-menopausal patients with ASC-US were similar (*P* > 0.05). The detection rate of CIN2+ in HPV52 pre-menopausal patients was significantly higher than that in the post-menopausal patients (*P* < 0.05) (**Table 6**).

**Table 6:** Histopathologic results for pre- and post-menopausal women with ASC-US according to single HPV genotyping.

	No. of pre-menopause Cases			No. of post-menopause Cases		
	≤CIN1	CIN2+	Total	≤CIN1	CIN2+	Total
Total	748	209	957	319	48	367
HPV16	131	96^b^	227	45	23	68
HPV52	154	35^a^	189	61	4	65
HPV58	94	19 ^b^	113	52	10	62
HPV18	48	16 ^b^	64	19	1	20
HPV33	37	19 ^b^	56	17	4	21
HPV53	45	2	47	25	0	25
HPV39	36	6	42	12	0	12
HPV56	38	2	40	20	0	20
HPV31	29	8	37	11	4	15
HPV68	36	0	36	7	0	7
HPV51	31	1	32	13	2	15
HPV59	23	2	25	18	0	18
HPV66	25	0	25	12	0	12
HPV35	10	2	12	2	0	2
HPV45	6	0	6	3	0	3
HPV73	3	0	3	0	0	0
HPV82	2	1	3	2	0	2

a P < 0.05 compared with the post-menopause counterpart groups.

b P ≥ 0.05 compared with the post-menopause counterpart groups.

### 3.3 Vaginal microecology analysis results for women with ASC-US

In total, 499 patients underwent vaginal microecology analysis among the 2,462 patients with ASC-US in this study. In general, the abnormal reporting rate for vaginal microecology was 64.3% (321/499). The prevalence of abnormal reporting was significantly lower in the pre-menopausal group (56.2%) than that in the post-menopausal group (82.9%; *P*<0.05; **Table 7**). The rate of vaginal microecological abnormalities in the HR HPV-positive patients with ASC-US (64.3%) was similar to that in the HR HPV-negative patients with ASC-US (64.6%; *P*>0.05).

**Table 7:** Vaginal microecology analysis results for women with ASC-US according to menopause state and HPV result.

	No. of Cases (%)					
		Pre-menopause	Post-menopause	HPV(+)	HPV(-)
	Total	347	152	420	79
Vaginal microecology analysis	normal	152(43.8)	26(17.1)	150(35.7)	28(35.4)
	Abnormal	195(56.2)	126(82.9)	270(64.3)	51(64.6)

In the present study, the abnormal vaginal microecology rate of BV was the highest, accounting for 18.6% (188/448). However, the distribution of vaginal microecological abnormalities was different in the pre- and post-menopausal groups, and the prevalence of BV (19.6%), BV intermediate type (9.8%), and mixed vaginitis were relatively high in the premenopausal group, but abnormalities in the bacteria-inhibiting flora were mainly evident in the post-menopausal group, accounting for 40.8% (**Table 8**). There was no significant difference in the prevalence of BV, leukocytosis, intermediate type BV, trichomoniasis, and mixed vaginitis between the pre-menopausal and post-menopausal patients with ASC-US (*P* > 0.05). However, the prevalence of VVC in the pre-menopausal group was significantly higher than that in the post-menopausal group (*P*<0.05). The prevalence of AV, bacteria-inhibiting flora, and atrophic vaginitis in the post-menopausal patients was significantly higher than that the in pre-menopausal patients (*P* < 0.05).

**Table 8:** Vaginal microecology analysis Results for pre- and post-menopausal Women With ASC-US.

	No. of Vaginal microecology analysis Results (%)										
	normal	BV	VVC	AV	leukocytosis	BV intermediate type	bacteria inhibiting flora	Atrophic vaginitis	Trichomoniasis	mixed vaginitis	Total
Pre-menopause	152(43.80)	68(19.60)	22(6.34)	9 (2.59)	20(5.76)	34(9.80)	10(2.88)	2 (0.58)	2 (0.58)	28(8.07)	347
Post-menopause	26(17.11)	25(16.45)	1(0.66)	10(6.58)	3(1.97)	10(6.58)	62(40.79)	9 (5.92)	0	6 (3.94)	152
Total	178(35.67)	93(18.64)	23(4.61)	19(3.81)	23(4.61)	44(8.82)	72(14.43)	11 (2.20)	2 (0.40)	34(6.81)	499
*P*	<0.05	>0.05	<0.05	<0.05	>0.05	>0.05	<0.05	<0.05	>0.05	>0.05	

To further explore whether the vaginal microecological abnormalities can lead to a false positive diagnosis of ASC-US, we collected the vaginal microecological results of healthy women with negative HPV and NILM in the same period, including 92 pre-menopausal and 59 post-menopausal women(**Table 9**). The results showed that when HPV was negative, ASC-US was evident, and histopathology revealed chronic cervicitis, the microecological abnormality rate was 66.22% (49/74), of which the post-menopausal abnormality rate was 76.92% (20/26). The microecological abnormality rate of the normal control group was 52.32% (79/151), of which the post-menopausal microecological abnormality rate was 69.50% (41/59). The abnormal rate of vaginal microecology in the normal control group was significantly lower than that in the ASC-US group (*P <*0.048), and the false-positive rate of ASC-US diagnosis increased due to the abnormal microecology. However, there was no significant difference in the rate of microecological abnormalities between the post-menopausal and pre-menopausal women.

**Table 9:** Vaginal microecology analysis results for women with NILM and ASC-US.

Vaginal microecology analysis Results	NILM			ASC-US		
	Pre-menopause	Post-menopause	total	Pre-menopause	Post-menopause	total
Normal	54	18	72	19	6	25
Abnormal	38	41	79	29	20	49
Total	92	59	151	48	26	74

## 4 Discussion

ASC-US is the most common finding among the epithelial cell abnormality, but has an equivocal cytologic diagnosis. The histopathology results of ASC-US are very different and can either be an actively proliferated benign lesion or a potentially malignant lesion (Solomon et al., 2002).

ASC-US can be characterized by (1) squamous differentiation, (2) increased nuclear-to-cytoplasmic ratio, and (3) minimal nuclear changes that may include hyperchromasia, chromatin clumping, irregularity, smudging, and/or multinucleation. Subtle and subjective findings in the specimens with ASC-US have resulted in poor reproducibility, thus compounding the difficulty in developing and illustrating strict criteria. In the case of the presence of reactive/reparative or degenerative changes, organisms, air-drying with artifactual nuclear enlargement, atrophic patterns, and atrophy of other artifacts, it is difficult to diagnose NILM or ASC-US. Thus, referral to the patient’s age, medical history, and HPV results may be needed. In this study, the detection rate of ASCUS and the results of HPV, histopathology, and vaginal microecology were analyzed.

In this study, the detection rate of ASC-US was 5.7%, which is within the range of 3.7–10% of the ASC-US reporting rate in the Chinese population (Tao et al., 2015; Zheng et al., 2019; Guo et al., 2019; Tao et al., 2019; Tao et al., 2021). With age-stratified analysis, it was found that the detection rate of ASC-US in women aged 51–60, 61–70 and ≤20 years was higher than that in the women of childbearing age, which is compatible with the findings of Tao et al. (Tao et al., 2021). Women aged ≤20 years were not included in the range of cervical cancer screening according to the ASCCP guidelines. A relatively low number of women aged ≤20 years were screened, but the high detection rate of ASC-US may be due to the early age of sexual life and destruction of the cervical barrier caused by stimulation such as sexual intercourse. The average age of menopause in the Chinese women is 50 years old. In this study, the average age of menopause in the patients with ASC-US was 50.23 years old, which is comparable with the average age of menopause in the Chinese women (50 years). The estrogen levels decrease substantially with menopause, the basal layers of ectocervical epithelium become thinner, the proportion of immature basal and para basal cells increases, and the proportion of middle squamous cells decreases, which can lead to the relative enlargement of the nucleus, high nuclear–cytoplasmic ratio, hyperchromatic cells, and high nuclear–cytoplasmic ratio in the basal parathyrocytes. Many studies have confirmed that the “atrophic” form caused by low estrogen levels can increase the detection rate of ASCUS in the post-menopausal and elderly women (Cakmak and Köseoğlu, 2014).

In this study, the histopathological correlation indicated that CIN2+ and CIN3 were identified in 18.2% of the women who had ASC-US cytology. In a pooled analysis, the CIN2+ detection rate of ASC-US was 3.2% in the women aged 15–59 for initial screening, but the risk ratio greatly increased with the increase in the cumulative time and in the participants in the clinics or hospitals (Pan et al., 2014). A study conducted in the rural areas of Shanxi, China, reported that the detection rates of CIN2+ and CIN3+ in the patients with ASC-US were 7.28% and 1.75%, respectively (Wang et al., 2021). Through a systematic review, Pan et al. demonstrated that the detection rate of CIN2+ in the patients with ASC-US was 10.1% (Pan et al., 2020). Tao et al. investigated the detection rate of CIN2+ in the Chinese women with ASC-US and reported that CIN2+ was detected in 7.1% of the women with ASC-US among which 0.6% had cervical cancer (Tao et al., 2021). Similar to the observations from two other studies conducted in the United States, they found that the detection rate of cervical precancerous lesions in the patients with ASC-US was 5.1–9.7% (Stoler et al., 2011; Stoler et al., 2013). In this study, the high detection rate of CIN2+ in the patients with ASC-US may be attributed to several reasons. One reason is that some patients with ASC-US and having a HPV-negative status were not referred for colposcopic biopsy; therefore, there was an increase in the abnormal biopsy rate. Second, a small number of patients in this study had persistent ASC-US, which also increased the detection rate of CIN2+. In addition, the women with ASC-US reported in the previous studies were mostly of childbearing age or under 65 years of age. The age range in this study was large, and the rate of post-menopausal women with ASC-US was high, which also increased the detection rate of CIN2+. However, the relationship between ASC-US and histopathology in post-menopausal women remains controversial. Massad et al. reported that the women with ASC-US over 50 years of age had a similar detection rate of CIN2+ (about 11%) compared with those under 35 years of age, but the incidence of CIN3 and cancer was significantly higher in the older group (Massad et al., 2003). Tai et al. reported that the incidence of CIN3+ lesions in the women aged over 60 years was higher than that in the women aged between 30 and 60 years (Tai et al., 2018). Similar to other studies, the detection rate of CIN2+ in the post-menopausal women in this study was significantly lower than that in the pre-menopausal patients with ASC-US.

In the recent years, HR-HPV testing has been included in cervical cancer screening programs, which can be used to triage patients with ASC-US. According to the most current ASCCP risk-based management consensus guidelines, the immediate risk of CIN3+ is 4.4% for ASC-US/HPV-positive and 0.04% for ASC-US/HPV-negative populations (Perkins et al., 2020). Zheng et al. found that the CIN2+ lesions in 13.98% (124/887) of the women with ASC-US/HPV+ were significantly higher than those in the 2.84% (29/1022) of women with ASC-US/HPV-. They also found that cervical cancer was detected in 3.95% (35/887) of the women with ASC-US/HPV+ in the largest pathology laboratory in China (Zheng et al., 2019). The survey results of Tao et al. indicated that CIN2+ lesions were found in 657 (10.7%) of 6,154 HR-HPV-positive women with ASC-US compared with only 1.5% of HR-HPV-negative (Tao et al., 2021). In this study, CIN2+ and CIN3+ lesions were found in 21.5% and 4.6% of the women with HPV-positive/ASC-US, respectively, which were significantly higher than the women with HPV-negative/ASC-US (5.4% and 1.2%, respectively). This strongly supports the 2019 ASCCP guideline of reflex HPV testing for women with ASC-US cytology (Perkins et al., 2020). However, the risk of CIN2+ is different for the women with ASC-US/HPV+ of different ages. Wang et al. showed that in the women with ASC-US/HPV+, the risk of HSIL+ in the ≤30 years old group (40.52%), 31–40 years old group (39.67%), and 41–50 years old group (34.22%) were significantly higher than that in the 51–60 years old group (21.65%), but the risk of cervical cancer was significantly higher in the women >50 years old (Wang et al., 2020a). Feng et al. reported that the risk of CIN2-3 in the perimenopausal and post-menopausal women was lower than that in pre-menopausal women with ASC-US/HPV+ (Feng et al., 2008). Similar to these investigations, in our study, the detection rate of CIN2+ in the pre-menopausal women with ASC-US/HPV+ was significantly higher than that in the post-menopausal patients, but the rates of CIN3+ and cervical cancer in the pre- and post-menopausal women were similar. Furthermore, there was no significant difference in the detection rates of CIN2+ and CIN3+ in the pre- and post-menopausal women with ASC-US/HPV-.

According to the principle of “equal management of equal risk”, patients with ASC-US with different HPV genotypes should be treated differently in the clinical management. A systematic review conducted by Bonde et al. demonstrated that published evidence supports the clinical utility of HPV genotyping in risk discrimination for CIN3+ lesions during cervical cancer screening in the US, United Kingdom, Sweden, Denmark, and the Netherlands (Bonde et al., 2019). Recently, there is some experience using HR-HPV genotyping in triaging Chinese women with ASC-US.Cao et al. (2019) investigated the role of HPV 16/18 genotyping in 329 Chinese women and found that the sensitivity and specificity for HPV16/18 in detecting CIN2+ lesions in women with ASC-US were 82% and 91%, respectively (Cao et al., 2019). Wang et al. carried out a survey in the rural Shanxi province and found that compared with the 15 HR-HPV tests, genotyping for a combination of HPV16, 18, 31, 33, and 58 significantly increased the specificity with virtually no loss of sensitivity for detecting the CIN2+ and CIN3+ lesions. In addition, during the 2-year follow-up period, women with HPV16, 18, 31, 33, or 58 genotypes were the most likely population (92%, 23/25) to develop CIN2 lesion) (Wang et al., 2021). Pan et al. reported that the top five genotypes with respect to prevalence and risk of CIN2+ were HPV16, 58,18, 33, 31, and 52. The HPV16, 18, 31, 33, 52 and 58 model achieved higher sensitivity (91.3%) and specificity (70.0%) for the triage of patients with ASC-US than the other HR-HPV-type combination models (Pan et al., 2020). In the current study, we observed that the CIN2+ detection rate of HPV16/18 positive patients with ASC-US was significantly higher than that of the other high-risk HPV-positive groups, and the five highest-risk HPV genotypes were HPV16, 52, 58, 18, and 33. The five most common HPV genotypes (HPV16, 52, 58, 18, and 33) identified more CIN2+ lesions than the other HR HPV genotypes. Consistent with the results of several large studies in China, HPV16 was the most common genotype, while HPV52 and 58 were more common in the Chinese population than HPV18 (Wang et al., 2018; Zhao et al., 2018). Although HPV52 and 58 genotypes were more common than HPV 18, our results showed that the distribution of HPV genotypes in ASC-US was similar in the pre- and post-menopausal women. The most common HPV genotypes were HPV16, 52, 58, 18, and 33.

The vaginal microecology is a significant niche of the human microbiome, and the vaginal microbiota plays a significant role in the wellness maintenance of the female reproductive tract. When the vagina cannot maintain its microecological balance, the acidic environment of the vagina is destroyed, and the proliferation of pathogenic bacteria replaces the initial dominance of *Lactobacillus*. The increased diversity of the bacterial community may cause upper genital tract pathological infections. Cervicitis and vaginitis have been reported as major confounding factors for the diagnosis of squamous epithelial cell abnormality (Da Silva et al., 2013). To explore the association between lower genital tract infection and cervical cytological abnormalities, Liu et al. reported that in addition to BV, Trichomoniasis, and VVC, which are the influencing factors of low-grade lesions in cells, HR-HPV infection and microecological abnormalities were mainly related to ASC-US and LSIL. In HR-HPV-negative cases, vaginal microecological abnormalities mainly caused mild morphological changes and did not lead to further cancerous changes (Liu et al., 2021). Paba et al. demonstrated that microecological abnormalities may increase the diagnosis of ASC-US and suggested that the patients with microecological abnormalities should be treated before screening for HPV and cytology (Paba et al., 2015). Our study supports that vaginal microecological abnormalities are associated with the cytological diagnosis of ASC-US.

Overall, in our study, BV was the most common microecological abnormality, which is in agreement with the previous literature (Mitra et al., 2016; Liu et al., 2021). However, the types of microecological abnormalities were different in the pre- and post-menopausal women with ASC-US, and the prevalence of the BV and BV intermediate types was relatively higher in the premenopausal women. Post-menopausal microecological abnormalities were mainly attributed to the bacteria-inhibiting flora, which accounted for 40.8% of the total cases. Furthermore, the microecological abnormality rate of post-menopausal women with ASC-US was significantly higher than that of the premenopausal women. This may be due to the reduction in the estrogen levels in the post-menopausal women, which results in decreased root colonization by *Lactobacillu*s and facilitates the survival of pathogenic bacteria and viruses (Maturana et al., 2007). In addition, atrophy of the cervical epithelial cells and the high nuclear-to-cytoplasm ratio in the post-menopausal women make it difficult to distinguish them from normal cervical intraepithelial lesions. Therefore, we suggest that when cytology diagnoses ASC-US, attention should be paid to the detection of vaginal microecology. Premenopausal women should pay attention to the standard treatment for vaginitis and reduce the inflammation caused by infection to interfere with the diagnosis of ASC-US. After excluding the taboos associated with the use of estrogen, the local use of estrogen in the post-menopausal women can be suggested to reduce the shape of epithelial atrophy, and then review cytology. This can not only avoid the harm of false positives to the patient’s body, mind, and economy, but also avoid a missed diagnosis.

The strength of this study is that it is the first large-scale retrospective analysis to demonstrate differences in the distribution of high-risk HPV genotypes and histopathology for ASC-US in pre- and post-menopausal women. We also demonstrated an association between the vaginal microecology and ASC-US diagnosis. However, the current study had some limitations. First, this was a retrospective study, and not all women with ASC-US were referred for colposcopic biopsy. In our study, HPV positive/ASC-US represented the majority of the patients. Accordingly, the detection rate of CIN2+ was high, which could have introduced bias in the results. In addition, not all the women in the ASC-US group had vaginal microecology, and we did not perform a stratified analysis of the symptoms of the patients of vaginitis. In future studies, the influence of HPV should be excluded and the influence of vaginal microecology on the women with ASC-US/HPV should be analyzed. Second, we did not consider the impact of HPV vaccines in this study population. However, bivalent vaccines were only approved in China in 2016; therefore, the impact of vaccines in this study population is small, but it should be a focus for further stratified analysis in the future. Therefore, large-scale prospective studies are needed to verify the distribution of HPV genotypes in women with ASC-US, differences in ASC-US among the pre- and post-menopausal women, and the impact of vaginal microecology on the diagnosis of ASC-US.

In conclusion, the detection rate of ASC-US in women over 50 years old was higher than that in women less than 50 years old, but the detection rate of CIN2+ was lower in the post-menopausal women than in the premenopausal women with ASC-US. However, the vaginal microecological abnormalities may increase the false-positive diagnosis rate of ASC-US. The vaginal microecological abnormalities of menopausal women with ASC-US are mainly infectious diseases such as BV which occurs in post-menopausal women and are mainly caused by the decrease in the bacteria-inhibiting flora due to mucosal epithelial atrophy. Therefore, more attention should be paid to the detection of vaginal microecology to avoid the high referral rate for colposcopy.

## Data availability statement

The raw data supporting the conclusions of this article will be made available by the authors, without undue reservation.

## Ethics statement

The studies involving human participants were reviewed and approved by Ethical Committee Tianjin Medical University General Hospital Tianjin, China. Written informed consent for participation was not required for this study in accordance with the national legislation and the institutional requirements.

## Author contributions

AF, and FX conceived the study question, and all authors were involved in the study design. YY was involved in the statistical analysis while BL, LD, and CW interpreted the results. BL created the first draft of the manuscript. BL, LD, CW, JL, XZ, AF, and FX made substantial contributions to drafting the article and revising it critically. All authors listed have made a substantial, direct, and intellectual contribution to the work and approved it for publication.
